# Extending the spectrum of *AKT1* mosaicism: not just the Proteus syndrome

**DOI:** 10.1111/bjd.14478

**Published:** 2016-06-23

**Authors:** S. Polubothu, L. Al‐Olabi, L. Wilson, W.K. Chong, V.A. Kinsler

**Figure 1:**
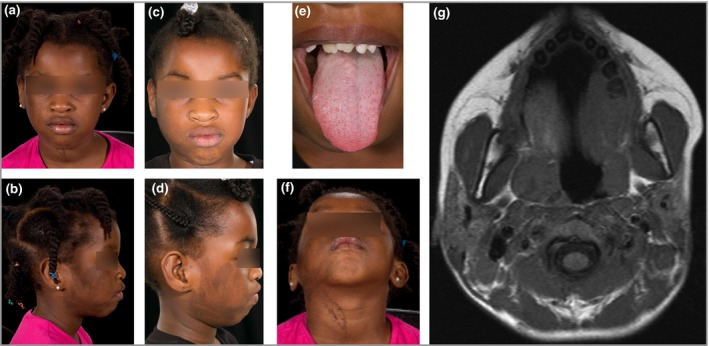
Increasing right‐sided soft tissue growth over time. (a, b) Initial presentation and (c, d) months later. (e) Right‐sided hypertrophy of the tongue. (f) Epidermal naevus. (g) Axial T1‐weighted image at the level of the nasopharynx showing asymmetry of the facial soft tissues, mandible, parotid glands and tonsils, larger on the right side.

**Affiliations:** ^1^ Guy's and St Thomas’ NHS Foundation TrustLondonU.K; ^2^ Genetics and Genomic MedicineUCL Institute of Child HealthLondonU.K; ^3^ Clinical GeneticsGreat Ormond St Hospital for ChildrenLondonU.K; ^4^ Department of RadiologyGreat Ormond St Hospital for ChildrenLondonU.K; ^5^ Paediatric DermatologyGreat Ormond St Hospital for ChildrenLondonU.K

dear editor, A 5‐year‐old girl was referred to us for evaluation of a pigmented birthmark, unchanged since birth, on her right cheek. There was no other previous medical history of note and no family history of relevant problems. Cutaneous examination revealed a pigmented keratinocytic epidermal naevus following fine Blaschko lines on the right cheek and neck (Fig. 1), and a solitary, 1‐cm café‐au‐lait macule on the lower leg. Subtle hemihypertrophy of the right cheek was noted, including the tragus of the ear, which had appeared to progress over time. In conjunction, she was noted to have right‐sided hemihypertrophy of the tongue with intraoral pigmentation of the mucosal surfaces, linear naevoid‐looking change on the right side of the tongue and right‐sided misalignment of the teeth. Her parents also gave a history of frequent malodorous right ear discharge. The patient was referred to otorhinolaryngology, who diagnosed a severe right‐sided conductive hearing loss. The remainder of her physical examination was unremarkable. Magnetic resonance imaging confirmed soft tissue hemihypertrophy of the cheek, with extensive soft tissue overgrowth within the right external auditory canal and middle‐ear cleft, and a sclerotic bony spur in the posterior inferior aspect of the external auditory canal (Fig. 1g).

**Figure 2:**
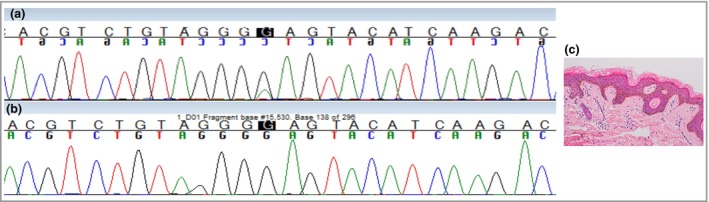
(a) Sanger trace showing heterozygous missense mutation c.49G>A, p.Glu17Lys in affected skin (epidermal naevus) but not (b) blood. (c) Histological section of epidermal naevus.

Mosaicism for a growth‐promoting mutation was suspected. With appropriate consent for genetic investigations a punch skin biopsy was obtained from the epidermal naevus, and DNA extracted directly by standard methods. DNA was also extracted from a venous blood sample. Histology confirmed a keratinocytic epidermal naevus (Fig. 2). Initial sequencing of *HRAS* and *KRAS* hotspot mutations, as described in epidermal naevus syndromes,^1^, ^2^ was negative. However, Sanger sequencing for hotspot mutations in *AKT1* revealed a heterozygous missense mutation, c.49G>A, p.Glu17Lys, present in the epidermal naevus but not the blood (Fig. 2). Although we only have a single biopsy from this patient, given existing knowledge of this particular mutation, postzygotic mosaicism is highly likely to be the cause of her entire clinical phenotype and not just the epidermal naevus.

In mosaic form, this is the characteristic causal mutation in the first cohort of patients with clinically delineated Proteus syndrome, as defined by strict diagnostic criteria.^3^, ^4^ Proteus syndrome is a rare overgrowth syndrome characterized by a normal phenotype or limited features at birth followed by progressive disproportionate, asymmetric overgrowth with skeletal defects, dysregulated adipose tissue, pulmonary abnormalities, and a propensity to deep vein thrombosis and pulmonary embolism. Patients may have dysmorphic facies, intellectual disability and, in some cases, seizures and brain malformations. Cutaneous lesions associated with Proteus syndrome include epidermal naevi, vascular malformations, lipomas and the characteristic cerebriform connective tissue naevus.

In 2001, Lindhurst *et al*. identified somatic activating mutations in *AKT1* as the cause of Proteus syndrome,^3^ discovering the *AKT1* mutation c.49G>A, p.Glu17Lys in the affected tissues of 26 of 29 patients who met the clinical diagnostic criteria for Proteus syndrome. Activating mutations of the *AKT1* oncogene leads to Akt kinase activation, triggering the PI3K/Akt/mammalian target of rapamycin pathway, a critical pathway in the regulation of cellular proliferation and apoptosis. From these published cases we can clearly see that a clinical diagnosis of Proteus syndrome is likely to be caused by *AKT1* mutation mosaicism in most, if not all, cases; however, it is now also evident that the reverse is not necessarily true.

This case depicts the most restricted phenotype of *AKT1* mosaicism, confirmed by sequencing, described so far, limited, presumably, by the timing of the postzygotic mutation and the cell type affected. This mirrors the spectrum of severity seen in other known mosaic disorders involving oncogenes and tumour suppressor genes, such as congenital melanocytic naevus syndrome and the *PIK3CA*‐related overgrowth spectrum (PROS) disorders.^5^, ^6^ Suspected cases of ‘mild’ Proteus syndrome have previously been described in the literature, prior to the discovery of the underlying genetic mutation.^7^, ^8^, ^9^, ^10^ Although, without molecular confirmation the description of many of these cases may similarly represent milder phenotypes of other segmental overgrowth syndromes, for example the *PIK3CA*‐related overgrowth spectrum. One previous case of a limited Proteus phenotype, and corresponding *AKT1* mosaic genotype, has been described in a 33‐year‐old man presenting in childhood with bilateral plantar cerebriform collagenomas.^11^ Similar to our case, this case does not meet the established clinical diagnostic criteria for Proteus syndrome; however, the presence of the characteristic cerebriform collagenomas could alert clinicians to the possibility of more restricted *AKT1* mosaicism. Our case extends this spectrum into the much milder phenotype of epidermal naevus with underlying overgrowth. Clinicians should be alerted to this restricted phenotype and consider *AKT1* screening in undiagnosed cases of mild overgrowth in association with correlating cutaneous lesions, and also as part of genetic investigations for epidermal naevi.
